# A relational conceptual framework for multidisciplinary health research centre infrastructure

**DOI:** 10.1186/1478-4505-8-29

**Published:** 2010-10-06

**Authors:** Stephanie E Coen, Joan L Bottorff, Joy L Johnson, Pamela A Ratner

**Affiliations:** 1 School of Nursing, University of British Columbia, 302-6190 Agronomy Road, Vancouver, British Columbia, V6T 1Z3, Canada; 2 Institute for Healthy Living and Chronic Disease Prevention, University of British Columbia Okanagan, 3333 University Way, Kelowna, British Columbia, V1V 1V7, Canada

## Abstract

Although multidisciplinary and team-based approaches are increasingly acknowledged as necessary to address some of the most pressing contemporary health challenges, many researchers struggle with a lack of infrastructure to facilitate and formalise the requisite collaborations. Specialised research centres have emerged as an important organisational solution, yet centre productivity and sustainability are frequently dictated by the availability and security of infrastructure funds.

Despite being widely cited as a core component of research capacity building, infrastructure as a discrete concept has been rather analytically neglected, often treated as an implicit feature of research environments with little specification or relegated to a narrow category of physical or administrative inputs. The terms research infrastructure, capacity, and culture, among others, are deployed in overlapping and inconsistent ways, further obfuscating the crucial functions of infrastructure specifically and its relationships with associated concepts.

The case is made for an expanded conceptualisation of research infrastructure, one that moves beyond conventional 'hardware' notions. Drawing on a case analysis of NEXUS, a multidisciplinary health research centre based at the University of British Columbia, Canada, a conceptual framework is proposed that integrates the tangible and intangible structures that interactively underlie research centre functioning.

A relational approach holds potential to allow for more comprehensive accounting of the returns on infrastructure investment. For those developing new research centres or seeking to reinvigorate existing ones, this framework may be a useful guide for both centre design and evaluation.

## Background

Multidisciplinary approaches are increasingly acknowledged as necessary to address some of the most urgent contemporary health challenges, yet many university-based researchers struggle with a basic lack of administrative and material resources extending beyond departmental bounds to facilitate and formalise these collaborations. The highly decentralised structure typical of many university environments has traditionally circumscribed the scope of research support and facilities to specific faculties, schools, or departments. Such fragmentation creates major practical obstacles for investigators attempting to develop partnerships and teams with diverse expertise [1-3]. One organisational solution widely lauded for facilitating multidisciplinary collaboration is the extra-departmental research centre [2,4-11]. The primordial purpose of the research centre is "to do what departments cannot do: to operate in interdisciplinary, applied, or capital-intensive areas in response to social demands for new knowledge" (p. 17) [[12], see also [6,7,9,11,13]]. In this niche role, research centres are uniquely positioned to tighten the knowledge-to-action gap.

The transformative potential of a research centre, however, may be circumscribed by certain intrinsic structural limitations. Centre design is more often ad-hoc than theory-informed or evidence-based [6,13]. Such "erratic" organisational qualities may be at odds with the benefits of the flexibility and responsiveness afforded precisely by the very uniqueness of centre forms (p. 1) [6].

Indeed, what might be routinely branded research centres are often highly heterogeneous entities [5,6,13-16]. Friedman and Friedman described the organised research unit as "a nondepartmental structure variously termed...depending on the local administrative taxonomy" (p. 27) [5]. Similarly, Tash, drawing on survey data from 300 research consortia in the United States, reported that the labels institute, centre, and laboratory were used interchangeably, even though their attributed meanings were inconsistent and context-contingent [11]. Hence, arguments have been put forth for distinctions to be made among centres, units, and institutes [16]. Various typologies also have been tendered that characterise research organisations according to degrees of institutionalisation [17], linkages with funders, departments, or other sectors [11,12], types of collaborative arrangements [18], and resources, protocols, and goals [7]. With the proliferation of virtual research teams and remote collaborations independent of shared workspace or geographic location, the forms assumed by research centres are becoming even more varied [19-21]. A recent study of 604 research centres in US medical schools found that physical space was not a significant determinant of centre size or status [8]. It is perhaps not surprising then that, among this miscellany, funding has tended to favour scientific appeal over organisational soundness [13].

Research centres frequently rely on a limited pool of competitive term-specific, cyclical grants, often from a single source and narrowly designated for specific resource and administrative costs. As a result, they are highly susceptible to funding instability [9,10,15]. Assessing the progress of Canadian healthcare policy research centres, Mekel and Shortt concluded that funding insecurity leads to disproportionate investment of centre resources into survival manoeuvres, such as undertaking renewal applications and searches for new funding sources, leaving fewer resources available for other centre endeavours. The prospects become particularly grim for activities that may not be directly funded, such as knowledge translation (KT) [15]. Funding insecurity also can lead to 'mission drift' whereby funds outside the scope of the centre are pursued in the effort to maintain functionality [11,22]. Langille et al., in their appraisal of health promotion centres in a region of Canada, even cited infrastructure self-maintenance as one of six primary centre roles [22]. These inefficiencies ultimately hamper centre productivity and the capacity to generate innovative, applicable health evidence [14,15].

Further complicating this funding conundrum, traditional return on investment rubrics, to which granting agencies often subscribe, do not map on well to the multidisciplinary research centre for several reasons. First, the shear diversity of centre types complicates the application of any uniform approach [5,6]. Second, performance-based modes of evaluation enumerate discrete products, such as publications, and are thus ill-equipped to contend with time, variability, and context [14,23]. Interdisciplinary research, often requiring greater time inputs, is especially disfavoured by such measurements [1,3]. Bozeman et al. aptly pointed out that, in performance-based models, "the tendency to have science and technology products disembodied from the individuals and social context producing them provides an unrealistic overlay to evaluation" (p. 718) [23]. Third, by often rewarding a 'sure thing', performance-based criteria may lead to research 'homogenisation', discouraging innovation and experimentation even when the prospective societal benefits are great [24] - a course clearly counter to the chief advantage of research centres. Geuna and Martin went so far as to argue that the initial benefits of performance-based research funding systems result in longer term diminishing returns as contenders learn to 'play the game' [24].

Despite being centre stage in research centre funding and approaches to research capacity building [25-27], we contend that infrastructure as a discrete concept has been rather analytically neglected, often treated as an implicit feature of research environments with little specification or relegated to a narrow category of physical or administrative inputs. When specified, infrastructure is often uncritically consigned to readily apparent features, such as space, equipment, and research support staff [2,16,27-29]. Interestingly, these same discussions have pointed to the more dynamic and interactive potential of infrastructure, but have stopped short of detailing what such configurations might look like [cf. [25,29]]. The terms research infrastructure, capacity, and culture, among others, are also deployed in overlapping and inconsistent ways, further obfuscating the crucial functions of infrastructure specifically and its relationships with associated concepts [27].

In broader examinations of research environments, infrastructure is commonly included as one of many parsed features, separated from dimensions such as culture, visibility or identity, partnerships and linkages, training and mentorship, communications, and KT [2,22,25,27-29]. These divisions are helpful to the extent that they may delineate key areas for development or evaluation, but they also reinforce the notion that such aspects operate separately and that their outcomes are potentially measurable independently. Rather, we argue that compartmentalised approaches to research environments are largely limited by their inability to account for the *relationships *among components. Some research has supported the notion that interactions among features of research environments can yield concrete benefits. For example, Hanney et al., in their evaluation study of two health research centres in the United Kingdom, observed what they called the 'centre effect': research outputs generated circular returns to research centres by expanding their 'internal knowledge reservoir', which could be drawn upon, for example, in developing policy-relevant evidence. A strong centre identity also was shown to be beneficial in leveraging funds and other returns [14]. Descriptive and evaluative accounts of research centres also have made more ancillary observations regarding such potential interrelationships. In describing the strategies engaged by an academic nursing centre to increase productivity, Conn et al. remarked that beyond the designated purposes of the activities they undertook, certain initiatives also helped to enliven the local research culture [28]. Similarly, in their reflections on developing geriatric nursing centres of excellence, Beverly et al. noted that centre partnerships and skill-building programming served to boost creativity and propel research in new directions [30]. Huba et al., reporting on an evaluation of the aforementioned centres, recognised that there was a 'blended' quality to centre activities, in that they were multipurpose and addressed multiple outcomes [29]. We question whether such interactions may partly explain why research centre evaluation has proven problematic in many cases [6,9,15,24]; there is value added by these relationships that may go undetected or underestimated.

We suggest that some of the difficulty in articulating these interrelationships may be rooted in the conflation of the contextual or structural features of research environments and the research outcomes that they support. In some instances, we find that discussions of research centres often string together a range of traits or activities that are arguably of different orders. That is, areas that might be *outcomes *of research centre activities are aligned with elements that might be conceived as centre *structures *or resources to support the research process. For example, although Huba et al. acknowledged the multiple effects of centre activities, the key domains of enhancement they examined spanned from those that might be more structural, such as meetings, trainings, and workshops, to what might be considered intermediate outcomes, such as leveraging, and possibly higher order outcomes, such as improvements in patient care [29]; yet, no meaningful distinctions were made. In her framework for evaluating research capacity building, Cooke, while attempting to move beyond outcome measures and to include process indicators, similarly incorporated structural elements such as linkages and partnerships along with more outcome-oriented dimensions, such as dissemination [25]. Langille et al., likewise, included 'acquiring funds' among their otherwise categorically different essential research centre roles, such as communications and KT [22]. This enmeshment of structures and outcomes encumbers our potential to unpack the recursive 'centre effect'. This is not to say that outcomes do not reinforce or shape structures; certainly they are linked. The distinction we hope to draw is between resources that support centre functioning (structures) and the outcomes resulting from the level of centre functioning achieved, such as the capacity to effectively leverage funds.

In this paper, we develop a heuristic device to help move beyond some of these limitations. Drawing on a case analysis of NEXUS, a multidisciplinary health research centre based at the University of British Columbia, Canada, we explore the potential for an expanded conceptualisation of research infrastructure, one that specifies its largely assumed qualities while extending to articulate the interactive relationships among the tangible and intangible systems and structures underlying centre functioning. Pincus et al., in their report on a set of geriatric health care research centres, coined the concept *centerischkeit *to describe a sense of "centeredness" that was cultivated through "*structures *that brought people together, gave them a sense of belonging and worked to hold them together" (p. 281, emphasis added) [10]. Despite not having a physical space, each centre achieved a degree of *centerischkeit *through a range of organizational leadership tactics and collaborative activities. Although Pincus et al. did not go into detail about the specifics of *centerischkeit*, we aim to build on their idea and make such structures explicit.

## The making of a multidisciplinary research hub: NEXUS

The origins of NEXUS lie in the long-standing collaborations of a group of eight investigators at the University of British Columbia. Their work intersected to examine the social contexts of health behaviour, a rapidly expanding field that relies on bridging concepts from the health and social sciences. The NEXUS collaborators brought together their respective expertise in nursing, medicine, psychology, history, statistics, sociology, geography, epidemiology, population and public health, and educational psychology. Having been extremely productive informally, the group explored ways to further advance their collective research agenda under the leadership of three co-directors. In 2003, the British Columbia provincial funding body for health research, the Michael Smith Foundation for Health Research (MSFHR), announced a new programme to fund research infrastructure to establish research centres. The awards were designed to increase the productivity and competitiveness of researchers in the province. Funding was targeted to common services, including personnel, which would enhance the research environment, build critical mass, and improve integration within the research groups.

Refining their vision, the investigators submitted a successful application for an initial five years of funding - later extended by one year - to create NEXUS, a multidisciplinary centre mandated to generate evidence to inform practice and policy through a critical analysis of the social contexts that create barriers to health, affect health seeking, and influence health system responses. The analytical lenses of gender, diversity, and place were adopted as the overarching model for the NEXUS research agenda. Organisationally, the centre took shape as what might be considered a hybrid of a co-located and virtual team [19]: research support staff, two of the three directors, and several investigators were situated in the same physical workplace, while the majority of investigators maintained offices in their home departments and universities. Intra-centre communications were a blend of virtual and face-to-face meetings.

In its six-year lifetime, NEXUS uniquely supported the research capacity of 32 investigators from over a dozen disciplinary perspectives spanning the health and social sciences and humanities and over 70 trainees. Investigators represented a diverse group of researchers, policy and programme experts, and healthcare service providers and clinicians. With less than $1 million (CAD) in infrastructure investment, the centre leveraged over $12 million (CAD) in nationally competitive grants in support of its nationally and internationally recognised programmes of research in tobacco and substance use, youth sexual health, rural and remote health, heart health, women's and men's health, and cancer prevention. Based on the cumulative evidence of this comprehensive work, NEXUS implemented KT initiatives to improve the health of communities in British Columbia and elsewhere. NEXUS members were frequently sought for practice and policy input, including the commissioning of special reports and media commentary.

Despite its achievements, NEXUS was forced to close abruptly in 2009 when its provincial funding source was discontinued in a period of economic recession. When attempting to articulate the extent of this loss to government and funding body representatives, it became rapidly apparent that the consequences were not neatly circumscribed within a definable radius of the initial funding received; rather, certain features of the centre had evolved far beyond the core dollars. Such structures were complex and interlocking, and had been cultivated through the additional inputs of time, creativity, and the reinvestment of learning; thus, accounting for their loss proved less clear-cut.

Drawing on our experiences with NEXUS and current literature, we propose a relational conceptual framework for research infrastructure that seeks to address and resolve these tensions. Our inductive analysis was carried out through a series of meetings held with the NEXUS directorate and research manager where we examined annual centre reports, feedback from trainees, and interviews with investigators. To further our analysis, we critically reflected on our collective personal experiences within NEXUS, compared and contrasted these data with the literature, and used diagrams to capture emerging themes and their relationships.

## Infrastructure: a relational conceptual framework

**Figure 1:**
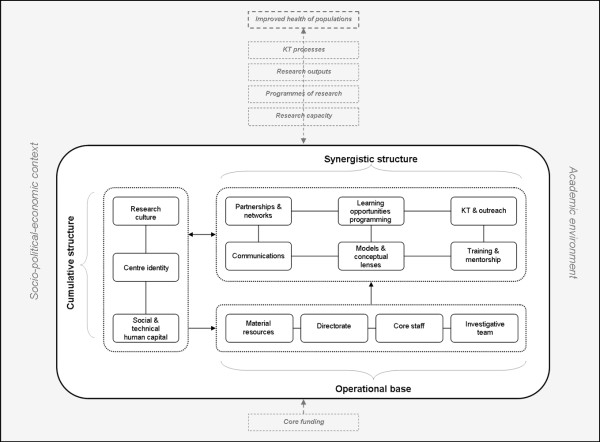
**A relational conceptual framework for multidisciplinary health research centre infrastructure.^§^**. ^§ ^Note: The contextual features and processes noted in the shaded area of the figure are indicated only insofar as to situate the roles of infrastructure within a broader context, and to acknowledge that infrastructure development is shaped by a range of factors. This model does not endeavour to address the intricacies of these complex external factors.

The conceptual framework we lay out is anchored in the contention that infrastructure consists of various structures that *interactively *create a composite greater than the sum of its parts (see Figure 1). We thus characterise this framework as *relational *because it is the relationships between these elements that define the whole, not simply the ingredients called for in the recipe. When focusing on interrelationships what becomes clear is that particular features of research centres take form as a result of an iterative, interactive process, and this form is not necessarily tangible. Instead, we see that more abstruse collective structures, such as internal knowledge, culture, or identity - that may not be quantifiable - are nevertheless significant factors in centre success. This is precisely one of the core assertions of our framework: such features of research environments as culture or identity are in effect resources upon which centre members can draw in advancing the work. These aspects thus form part of the underlying structures (albeit social) that support research.

Our model accordingly operates under the assumption that structures need not necessarily be material in nature to exert concrete consequences; they may also be the cumulative products of structure interactions that, in turn, feed back into the infrastructure montage. This broader approach permits the valuation of infrastructure components that may not be readily apparent in isolation; rather, once situated in relational context, we gain a wide-ranging picture of the interconnected and varied elements that underlie the collective functioning of the centre. From this perspective it is possible to more comprehensively account for the potential benefits of infrastructure funds, an important advantage for investigators drafting centre designs and for funders seeking to gauge the impacts of their investment.

While our framework problematises the structures *internal *to research centres, these clearly do not operate in isolation of wider contextual factors. Research centres are situated within particular academic environments within particular places with varying political, social, and economic climates - which is also why there are limits to the generalisability of this framework. We acknowledge these external factors, but fully exploring these is outside the scope of this paper (see Figure 1). In the following sections we describe the rationale and functions of each of the structural nodes - operational, synergistic, cumulative - and their various substructures in our framework, punctuating these with examples from NEXUS and the literature where relevant.

### Operational base

Borrowing a term used by Langille et al. in relation to Canadian health promotion centres [22], we refer to the foundational layer of our framework as the 'operational base'. The operational base consists of the primary material and human resources without which a centre would not exist. It is typically supported by a pool of core funding, often from external competitive sources, as well as possibly in-kind contributions from a home university. The operational base necessarily sets the stage for the potential development of the other structural nodes in our framework; if the operational base is jeopardised by, for example, funding uncertainty, other elements of centre infrastructure may waver, or not even have the opportunity to develop. Thus, while a key side of our argument in this paper is that infrastructure is more complex than purely materialist definitions allow, there is a crucial level of resources required to initiate and maintain - at the very least - a basic level of centre functioning. Needless to say, productivity is suboptimal at such a minimum state [19].

The operational base can be thought of as consisting of several subcomponents. The directorate provides centre leadership, including governance and decision-making guidance. The investigative team consists of the chief or principal investigators who lead and perform the research. Material resources comprise the physical inputs, such as physical office space or cybertechnology, or equipment and supplies and services [2]. By nature, centres benefit from spin-off economies of scale in resource sharing [5,6,9,16,17]. In terms of human resources, core staff members provide research and administrative support, and may include a research manager to oversee project coordination and facilitate funding grant preparation, an administrator to streamline collaboration logistics, and an in-house statistician or other types of analysts or technicians to facilitate the work [2]. Research support enables investigators to allocate more time for research and dissemination thereby increasing centre productivity [2,28], but it may also allow a centre to pursue other objectives linked to its mandate. At NEXUS, for example, research support staff enhanced centre workflow by facilitating grant preparation and daily centre management, thereby releasing core investigators to concentrate on advancing the research agenda. In addition, they helped to develop and implement KT initiatives, learning opportunities programming, and training activities, among other endeavours. Together, these operational structures create a foundation for the intermediate layer of our framework, synergistic structure, to which we turn now.

### Synergistic structure

Synergistic structure consists of programming, linkages, and activities that may or may not be supported directly by core infrastructure funding. We characterise these features in structural terms because they serve to expand the resource pool from which a centre can draw in both undertaking research and fulfilling its mandate; they are routinised, embedded, and integral to centre functioning, and developed and improved upon over time. These structures are marked as *synergistic *because they collectively serve to cultivate a variety of interactions among centre members, as well as between the centre and wider groups and individuals. In describing the benefits of research centres, former director of the United States' National Science Foundation, Erich Bloch pointed to the "power of interaction" generated by centre activities such as seminars and journal clubs (p. 374) [31]. It is due to this interactive quality that the synergistic structure is positioned as an intermediate layer between the operational base and the cumulative structure; it performs a crucial liaising function by mobilising the material inputs availed by the operational base to nourish the broader collective attributes and development of the centre. As described above, the extent of synergistic structure achieved - and the resulting potential for cumulative structure - is ultimately determined by the strength of the operational base; a poorly functioning base cannot support enduring derivatives.

Although each of the synergistic components are discussed separately below, their outcomes are interdependent and multiple. Each element contributes in overlapping ways to the generation of horizontal benefits, in terms of synergistic co-structures dialectically shaping each other, and vertical benefits, by feeding into a centre's *cumulative *structure, addressed in the next section. Several authors writing about research capacity building and research centre development have anecdotally noted these links both within the synergistic stratum, such as partnerships stimulating the development of new learning opportunities [30], as well as into the cumulative sphere, such as training activities driving centre culture [28] or communications fostering centre identity [22].

#### Communications

Communications encompass the scope of strategies for internal centre engagement, as well as relations with the wider public, stakeholders, and media. The communications products of centres, such as websites, newsletters, press releases or briefings, and other electronic media [10,15,22,28], are important centre resources for broadcasting to outside audiences, as well as creating internal continuity and a sense of community. At NEXUS, regular newsletters, an active website profiling member and centre activities, and press releases, where relevant, were mainstays of centre communications that dually supported internal connectivity and external exposure. Newsletters and the website helped to promote NEXUS events and programming to NEXUS members and the public, to spotlight members' achievements and centre accomplishments to the scientific community and funders, and to maintain and create new links with trainee alumni or relevant organisations. In addition, the collaborative and supportive content and tone of NEXUS communications reinforced and nurtured a collaborative centre culture. This also solidified a shared identity among members and enhanced the NEXUS reputation externally. (For examples of NEXUS communications, please visit https://circle.ubc.ca/handle/2429/13805.)

#### Learning opportunities programming

The literature cites a wide range of research-related activities enacted to bring together centre members for both formal and informal exchanges, including seminars, retreats, institutes, journal clubs, and other symposia [2,4,9,10,30]. Such programming is beneficial in stimulating ideas for innovation and new research directions [cf. [9,30]] and to more broadly cultivate a particular research culture and centre identity [9], explored in-depth in the next section. Such activities become systematised as part of a mode of operation for a research centre by providing regular spaces for creativity and connectivity. Learning programming at NEXUS included a seminar series, annual conferences, research poster sessions, and other events. These activities provided opportunities for students, junior investigators, and more established researchers to interact, present, and share their research, to explore new research directions, and to advance and refine centre models and conceptual lenses (discussed below). Importantly, they also created a platform for the centre identity and culture to grow, adapt, and evolve along with the lifecycles of research projects and the ever-expanding team.

#### Training and mentorship

Training is particularly crucial in terms of its contributions to the development of in-house social and technical human capital and the transmission of learning and the values that contribute to centre culture [22,32], two dimensions of cumulative structure described below. Concretely, this component might include formal training, such as practical skill-building workshops or mock external review panels [2,10,22,30], informal mentoring by way of supporting junior investigators and fostering links between students and faculty [2,10,32], or tangible support such as seed funding [2,9]. Heitkemper et al., in exploring how the Center for Women's Health and Gender Research at the University of Washington (United States) was able to expand its interdisciplinary research, identified training and mentorship activities as key to their success [2].

Trainees at NEXUS benefited from access to tangible resources such as funding for specialised training or travel to present at scientific conferences, as well as investigator-led skills-based workshops, and the broader NEXUS learning opportunities programming detailed above. Member profiles on the NEXUS website and frequent reports of trainee successes in NEXUS newsletters helped trainees to further capitalise on their affiliation with the centre by providing outlets to communicate their research to various audiences. Importantly, several trainees later became members of the NEXUS investigative team, further strengthening the centre's operational base. In addition, many alumni maintained close relations with the centre in their subsequent positions with other community-based or research-oriented health organisations, helping to expand the scope of NEXUS synergistic structure by introducing other related groups into the collaborative fold.

#### Partnerships and networks

Partnerships and networks, formal and informal, are social structures that facilitate the undertaking of research, provide channels to new opportunities, and stimulate the development of other synergistic components [29,30]. Enduring over time, such relationships may become part of the permanent infrastructure of a centre [2,29]. This structure may be particularly important for enabling other synergistic features, such as KT initiatives that seek to reach audiences outside academia.

The partnerships and networks that NEXUS developed over time with other organisations and individuals - including NEXUS trainee alumni - created valuable channels for research collaborations and dissemination, as well as crucial research-policy and research-practice linkages. For example, based on a long-standing programme of NEXUS research on families and tobacco use, NEXUS pilot-tested, produced, and disseminated a resource to help co-habiting couples reduce or stop smoking in conjunction with government health agencies, local addictions research centres, pregnancy outreach programmes, and other organisations.

#### Knowledge translation (KT) and public outreach

Effective channels for KT and public outreach are essential for meeting the mandated objectives of many research centres and ultimately facilitating the all-important goal of translating research into impact. Approaches may include focusing on the development of best practices [2,29], directing research products toward non-academic communities [2,22,29], holding events and meetings to share research with target audiences [15,22], and engaging with the media [22]. These structures are formative of centre identity by increasing external visibility to diverse audiences [2].

KT initiatives and public engagement activities became integral assets of the NEXUS research environment, connecting NEXUS research with various audiences and knowledge stakeholders. For example, while individual projects within the centre frequently employed project-specific KT strategies to communicate research findings and develop evidence-based health promotion tools, the centre also held public events on topics of popular interest. These activities provided a valuable platform for public engagement with research and allowed NEXUS to demonstrate the relevance of its work, showcase a range of NEXUS projects, and helped to establish a public image, a point we come back to below. Partnerships and networks, as discussed above, as well as other synergistic features, such as communications, enabled these endeavours to be developed and successfully executed.

#### Models and conceptual lenses

Models and lenses are the conceptual glue of a centre, consisting of the overarching analytical approaches or perspectives adopted to determine what is in line with a centre's mandate and what is out. It is the models and lenses that add cohesion and guide centre projects and activities. They may be refined and adapted over time. Besides this primary role, models and conceptual lenses can be instrumental resources in themselves by helping to brand a centre. At NEXUS, the gender-place-diversity triad became a trademark of NEXUS research that helped to further position the niche socio-contextual focus of the centre within the health research community. As such, the NEXUS lenses were not only essential in advancing and unifying the NEXUS research agenda, but also became an important mechanism for enhancing external visibility and in establishing the NEXUS identity. Gender-place-diversity became a useful instrument that NEXUS investigators brought to bear in their discussions and work in other contexts.

### Cumulative structure

This summative layer of our framework is a *cumulative *product of the ongoing interactions of the operational base and synergistic structure over time; it cannot exist independently of these antecedent structures. Accordingly, cumulative structure is manifest only at the aggregate level and is not reducible to smaller scales or attributable to specific substructures; it is a property of the collective. In this way, cumulative structure can be seen as analogous to concepts developed and adapted to understand neighbourhood social structures and contextual effects, such as collective social functioning or social capital [33,34]. Cumulative structure is similarly imbued with a 'public good' quality in that its benefits are centre-wide [33,35]. NEXUS, for example, benefited from its cache of in-house expertise, a highly reputed group identity, and a research culture conducive to achieving centre objectives.

This cumulative node uniquely completes a valuable feedback loop in centre functioning: it is continually shaped by and shapes the preceding layers. It is through this iterative cycling that centre infrastructure is able to remain relevant and continue to meet evolving centre demands. Structures disconnected from these flows - such as a partnership 'out of sync' with centre culture - stagnate, and their utility is greatly diminished. In addition to these important recursive functions, these cumulative components are resources in their own right yielding concrete benefits in support of research outcomes, such as leveraging an affiliation with a high calibre centre in support of a funding application. Despite these crucial roles, it is precisely this type of infrastructure that is exceptionally difficult to accurately valuate given that it is interactively generated over time.

#### Research culture

We contend that research culture - the values and norms that both guide how research is undertaken and how components in the operational and synergistic levels are engaged - is a species of centre social structure. When positioned relationally with other infrastructure components, it becomes clear how research culture fits into the backbone of centre functioning. Synergistic components, such as seminars, provide the channels for reification of centre culture, which in turn shapes the nature of operational and synergistic development.

At a very practical level, research culture is a force shaping collaborative behaviour and thus the functioning of a centre [9,22,28]. Looking to the organizational behaviour literature, it is clear that in some cases culture is a source of sustained competitive advantage [36]. Indeed, organisational culture is postulated to affect the extent to which creativity and innovation are stimulated [37]. Learning to work together, the centre's *modus operandi*, is a feature of the centre itself, learned and developed over time through the overall compilation of centre undertakings. Another aspect of culture, however, is more difficult to pinpoint. According to Mallon, engaging the words of one of his interviewees, a key benefit of membership in a research centre can be best described as "a spiritual notion, a sense of creativity, of intellectual excitement, of 'the feeling that anything is possible'" (p. 506) [9]. Achieving such a stimulating culture is surely an important driver for innovative health research.

For NEXUS, a culture that promoted non-hierarchical collaboration, egalitarian and participatory decision making, and mutual support was integral to its success. Aspects of synergistic structure, such as training and mentorship - by for example being inclusive of trainees in the centre community and privileging junior investigators where possible - critically shaped this feature of the research environment.

#### Centre identity

According to Youtie et al., self-recognition or internal identification is one of the minimal conditions for a research centre [17]. Centre identity encompasses not only scientific reputation, credibility, visibility, and leadership, but also the shared sense of identity experienced by centre members. The types of activities - or rather synergistic structure - enacted by a centre may work to promote or consolidate a particular centre identity [2,14,29]. Time is also a key ingredient, as well as common ground or goals [19].

Centre identity is an especially valuable resource in the knowledge-to-action process; a high calibre research identity may afford researchers greater capacity to influence relevant stakeholders, and stakeholders may refer to the centre for expert consultation. An identity may also be advantageous for gaining access to new opportunities, such as prestigious positions [2,29]. A meritorious research identity may be favourably leveraged by centre members for tangible returns, including funding or awards, time, space, resources, or access to research participant pools [2,14,29]. At a more ethereal level, sharing in a particular research identity may provide researchers with a sense of belonging [9,10] or a 'ready-made affinity group' [9], effectively strengthening the collaborative project. In the case of NEXUS, for example, beyond the obvious benefits of affiliation with a well-reputed centre, the NEXUS identity helped to cultivate an intellectual home for a geographically and disciplinarily diffuse group of members.

#### Social and technical human capital

In addition to creating economies of scale for material resources, the organisational structure of a research centre also inherently creates economies of scale in knowledge and skills by way of bringing together unique combinations of expertise [4,5,17], with public good-like benefits [4]. This notion is echoed across the research centre literature, with many citing the benefits of concentrating knowledge and expertise [2,14,22,27,29,32] and social capital in research centres [22]. Bozeman and Corley in their work on developing evaluations for science and technology projects and programmes, advocate the concept of social and technical human capital to inclusively describe "the sum of skills, knowledge, and social relations needed to participate in science" (p. 601) [[4], see also [23]]. Social and technical human capital brings together the notions of social capital and human capital to provide a way to think about the various forms of capital harnessed by the collection of individuals within a scientific consortium, and how these operate at a contextual level. One of the key thrusts of this concept is that the scientific capabilities of specialised research centres cannot possibly reside within a singular scientist. As a collective research centre attribute, social and technical human capital is a vital structural component for advancing the research agenda of a centre. Particular types of collaborative research arrangements, including those that are interdisciplinary, have accordingly been shown to enhance social and technical human capital [13]. It is as sources of social and technical human capital that research centres may be well-angled to influence policy and practice [2]. The unique combinations of intra-centre skills and expertise at NEXUS undoubtedly enabled the expansion of lone-grant projects into long-standing programmes of research and the rapid mobilisation of centre personnel to develop new investigations in response to critical emergent research areas, and to respond to targeted or catalyst research funding opportunities.

## Conclusions

We have made the case in this paper for an expanded conceptualisation of multidisciplinary health research centre infrastructure, one that envisions infrastructure as a relational construct comprised of multiple and interactive parts. In the case of NEXUS, it is clear that a range of centre features together enabled the collaboration, innovation, learning, and excellence that characterised the high-level functioning of the centre. The structures comprising the NEXUS research environment were not mutually exclusive, but instead interdependent and overlapping in their contributions to centre success. By attending to these relationships, more comprehensive appraisals of the structures involved in supporting research are possible. While beyond the scope of this paper, there are a range of evaluation frameworks that may prove helpful in developing the metrics and understanding the levers of centre performance [cf. [24]]. We suggest that our framework provides valuable points of reference for centre evaluations that are more attuned to the processes involved in multidisciplinary health research and the micro social contexts in which it is conducted.

An important caveat about our framework is that there is clearly no 'one-size-fits-all' solution; strategies for centre success are context-contingent [17,27]. This paper is based on a particular case study and does not claim to be readily transposable across research environments, including biomedical settings. A critical angle for future empirical work will be to investigate the wider contextual factors that influence research centre life. The fate of NEXUS, for example, was dictated by a provincial government budget deficit and subsequent realignment of government spending priorities. Even though one key benefit of research centres is their capacity to react quickly to such contextual changes, including societal demands for knowledge and new funding opportunities [9,11,15], sustainability remains an ongoing dilemma [15]. Lack of formal exit plans may lead to centre inertia, whereby organisations continue to exist past their point of scientific and social relevance [5,9]. Such work might shed light on the developmental trajectories of research centres within specific research funding systems. Indeed, a key question moving forward will be how research centres can develop sustainability strategies that are contextually-sensitive, allowing creative options for existing infrastructure to be translated or transformed before it is altogether discarded.

A relational framework thus broadens the lens for viewing the elements underlying centre functioning and accommodates less traditionally considered compositions. First, conventional 'hardware' notions of infrastructure arguably favour biomedical requirements for expensive instrumentation and technology; multidisciplinary health research centres incorporating social science perspectives may not call for comparable physical inputs. By incorporating time-contingent components and social structures, our framework accounts for the complexities of multidisciplinary research settings in particular [3,23]. Second, with the increasing emergence of virtual research centres [9,19,20], our conceptualisation provides a way forward in thinking about centre infrastructure independent of co-located workspace and facilities. A chief strength of our framework is that it allows for more inclusive consideration of the diversity of forms that multidisciplinary research centres manifest, addressing a key limitation cited in critiques of centre design and evaluation. This expanded scope, as we have suggested here, opens the door for developing new models to inform the theoretical basis for centre design to help guide the development of new research centres or reinvigorate existing ones.

## Competing interests

The authors declare that they have no competing interests.

## Authors' contributions

All authors were involved in conceptualising the framework and contributed to the text of this paper. All authors read and approved the final manuscript.

## Authors' information

JLB, JLJ, and PAR were founders and co-directors of NEXUS. SEC was the research manager for NEXUS in the 2008-2009 period until its closing.
